# Meat Consumers’ Opinion Regarding Unhealthy Pigs: Should They Be Treated with Antibiotics or Euthanized on Farm?

**DOI:** 10.3390/antibiotics10010060

**Published:** 2021-01-09

**Authors:** Dayane Lemos Teixeira, Daniel Enriquez-Hidalgo, Tamara Estay Espinoza, Fernando Bas, Maria José Hötzel

**Affiliations:** 1 Instituto de Ciencias Agroalimentarias, Animales y Ambientales (ICA3), Universidad de O’Higgins, San Fernando 3070000, Chile; 2 Animal Welfare Program, IRTA, Monells, 17121 Girona, Spain; 3 Departamento de Ciencias Animales, Pontificia Universidad Católica de Chile, Vicuña Mackenna 4860, Macul, Santiago 7820436, Chile; tyestay@uc.cl (T.E.E.); fbas@uc.cl (F.B.); 4 Bristol Veterinary School, University of Bristol, Langford, Bristol BS40 5DU, UK; 5 Sustainable Agriculture Sciences Department, Rothamsted Research, North Wyke, Okehampton EX20 2SB, UK; 6 Laboratório de Etologia Aplicada e Bem-Estar Animal, Universidade Federal de Santa Catarina, Rod. Admar Gonzaga 1346, Itacorubi, Florianópolis 88034-001, Brazil; maria.j.hotzel@ufsc.br

**Keywords:** consumer, perception, antibiotic, opinion, pig, welfare, euthanasia

## Abstract

The aim of this study was to investigate the opinions of meat consumers (n = 1780) on on-farm management of unhealthy pigs, whether support for treatment with antibiotics varies according with chance of recovery, and the effect of knowledge on the use of antibiotics on these opinions. Most participants believed that the use of antibiotics was the best solution for unhealthy pigs, and this was associated with a low level of knowledge about antibiotics. Increasing the probability of recovery after treatment increased support for treating pigs with antibiotics. However, the majority of participants rejected the consumption of meat from animals housed in “hospital pens”. After price, concern with food safety was the second main factor that influenced participants’ choice when buying meat. Support for the use of antibiotics to deal with unhealthy pigs in “hospital pens”, as well as for consumption of the meat from these animals, was higher among participants involved in agriculture. This shows that consumers are unaware of the potential negative repercussions for animal welfare associated with banning or reducing the use of antibiotics in livestock production, which is an important concern for the industry.

## 1. Introduction

Animal products are an important source of nutrients for many people around the world; production of animal protein quadrupled over the past 50 years, and the global demand continues to grow [1]. Concomitantly, the way animals are raised on farms has become more intensive over recent decades [2], with increasing adoption of confinement systems [3]. Critics argue that some practices used in intensive systems can harm the environment, rural communities, worker safety, food quality, food safety and animal welfare [4].

Citizens are becoming increasingly aware and interested about the living conditions and the welfare of farm animals. Public rejection of some aspects of intensive animal production systems has led to the development of regulations and industry actions associated with animal care on the farm, during transport and at the abattoir [5]. Recent studies in developed and developing nations have shown that citizens are concerned about husbandry systems and animal welfare [6,7,8,9,10,11].

Consumers’ opinions are greatly influenced by perceptions of risk and ethical assessments, especially those related to animal welfare and human health [12]. Many have concerns about health hazards posed by the residues of chemicals, pesticides, antibiotics and hormones in food products [13,14,15,16]. These concerns are not unfounded. Antibiotics have been commonly used as prophylactic methods in many animal production systems and to promote animal growth [17]. Comparative analysis of the amounts of antimicrobials used in food-producing animals and in human medicine strongly suggests that the most important selective pressure for antibiotic resistant bacteria might be the excessive use of antibiotics in the livestock industry [18]. Antibiotic resistance is considered one of the greatest threats to public health and there is a need for urgent action [19]. Although there are concerns that restriction of the use of antibiotics in food producing animals may negatively impact animal health and welfare [20], the World Health Organization (WHO) recently undertook a rigorous process, evaluating international standards, to develop and publish formal guidelines on the prudent use of antimicrobials in food-producing animals [21].

Pork is one of the most popular meats in the world. As a consequence of the current intensive rearing systems, pigs have high susceptibility to pathogens and to welfare problems associated with housing, stockmanship and/or environmental factors [22]. Antibiotics are frequently used in intensive pig production [23], mainly because of the low space available, with poor air and a high level of pathogenic bacteria, which makes pigs prone to diseases and injuries. In intensive production systems, unhealthy pigs that have, or are suspected of having injuries or illnesses are usually housed in a place called a “hospital pen” to help improve their condition and/or to avoid infecting healthy animals. However, these animals tend to suffer from their injuries or diseases and are often treated with antibiotics. Additionally, they tend to present a high risk that part of their meat cannot be used, which represents an economic loss for the farm [24]. On-farm euthanasia is an alternative for sick/infected pigs in inevitable situations; however, the use of inadequate methods can cause pain and suffering, compromising their welfare [25]. Considering the potential impact of this production practice on public opinion, the image of the pork sector and mood of the stockpeople, the humane on-farm euthanasia of pigs is a subject of high debate within the pig farming industry [26,27].

The attitudes of pig farmers towards the use of “hospital pens” [28] and the attitudes of caretakers towards swine euthanasia [27] in intensive pig production systems have been previously investigated. However, the attitudes and opinions of consumers regarding euthanasia of pigs on the farm, the use of “hospital pens”, and the use of antibiotics to treat pigs that will be used in food production remain to be fully understood. Therefore, the aim of this study was to investigate the opinions of meat consumers towards the most appropriate solution for unhealthy pigs, their support for treatment with antibiotics for unhealthy animals with different probabilities of recovery after treatment and the influence of their knowledge about the use of antibiotics on their opinion.

## 2. Materials and Methods

### 2.1. Participant Recruitment

This study was carried out in Chile and consisted of a survey with 1827 participants. The survey used both face-to-face and online questionnaires, due to the outbreak of COVID-19. The face-to-face questionnaire was carried out in the cities of Santiago and Concepción, located in the Metropolitan Region and the Biobío region, respectively. Participants were recruited by personal invitation in public places such as parks, shopping malls and medical clinic waiting areas. Participants were asked if they would be willing to take a survey about pig production, without any other specification. The online version was collected via a platform (Google Drive, http://drive.google.com/drive/), circulated through social media outlets (e.g., Facebook and Whatsapp) and sent to email lists of different organizations. In both cases (personal and online recruitment), only Chilean citizens that were at least 18 years old and were meat consumers (beef, pork and/or poultry) were included in the study. The identity of participants was not required.

### 2.2. Description of the Survey

Data collection was conducted between January and May 2020. After the first 20 participants had completed the face-to-face questionnaire, the responses were reviewed and refinements made to the questionnaire.

There was no interaction between recruiter and respondent after the acceptance to participate in the survey, and the questionnaire was self-administered. Participants were invited to read a consent form and sign (written version) or accept (online version) it before taking the survey. Personally collected data were transcribed to the Google Forms used for the online questionnaire, and all information was automatically transcribed to a Microsoft^®^ Excel sheet for Mac 2011.

The questionnaire included a total of 11 closed questions. The first questions addressed participants’ socio-demographic information relating to sex (male or female), age (18–25, 26–35, 36–45, 46–55, 56–65, or over 65 years old), education (up to high school, or higher education (completed or on-going)), and their involvement in agriculture (not involved, professional involvement—rural producer, student, academic, etc., or “grew up in an agricultural environment” (not currently involved but family owned a farm or participated in some form of agricultural activity)). Participants were asked how important eating meat was for them as an individual (not important, indifferent, important) and which of the options most influenced their choice when buying pork, beef and/or chicken (animal welfare, food safety, sustainability, price, other—please specify). Participants were asked to complete a three-question knowledge quiz on the use of antibiotics and mode of action, with response options as “true”, “false”, and “I do not know”.

Participants were then asked to read a short text on unhealthy pigs:

*Pigs that have, or are suspected of having injuries or illnesses (e.g., diarrhoea or respiratory infections) are usually housed in a place called a “hospital pen”, isolated from healthy animals. The goal is to improve their condition and to avoid infecting healthy animals.*

*These pigs remain in the “hospital pen”, not only to allow them to recover but also because of the difficulty that producers have in correctly discarding the euthanized pigs on the farms.*

*The percentage of pigs that enter the “hospital pen” is very low, not exceeding 1% of the herd. However, these animals tend to suffer from their injuries or diseases, receive antibiotics, and have a high probability that their meat can not be used, representing an economic loss for the farm.*

Thereafter, participants were asked their opinion regarding this statement: (Q1) “Considering the information described above, which of the following options do you consider to be the most appropriate solution?” The options were “Euthanizing pigs on the farm at the time of diarrhea or respiratory infection diagnosis to avoid spreading the disease to healthy pigs”, “Moving sick pigs to the “hospital pen”, but not giving antibiotics”, “Moving sick pigs to the “hospital pen” and providing conditions that allow them to improve, including giving antibiotics”. They were then asked their opinion regarding the problem for the production system. (Q2): “The situation presented in the text is considered a problem for animal welfare and food safety. Which of the following aspects do you think is the most problematic in pig production?” The options were “Inappropriate or excessive use of antibiotics when raising pigs”, “Suffering of pigs with injuries or diseases, who should be immediately euthanized”, “Deficiency in waste management and difficulty in discarding euthanized pigs”, “other—please specify”. Participants were also asked if (Q3) they supported the use of animals that were housed in “hospital pens” for human consumption, with response options of “support”, “indifferent”, and “reject”.

Finally, participants were randomized into three treatment groups, which corresponded to different probabilities that an animal would recover after treatment: (Q4) “*On a farm, a pig was diagnosed with a disease. The responsible veterinarian estimates that the animal has an “X”%* (20%, 50% or 80%) *chance of recovering if it is treated with antibiotics. Which of the following options do you consider to be the most appropriate?*”, with options, “Applying antibiotics in an effort to recover this animal that will be used for food production”, “Euthanizing this pig on the farm after the disease is diagnosed”, “other—please specify”.

### 2.3. Statistical Analysis

From the initial 1827 participants, 41 were excluded because they did not fit the pre-established requirements and 6 were excluded for miscellaneous reasons, resulting in 1780 usable questionnaires. Descriptive statistics for the responses were calculated using Microsoft^®^ Excel for Mac 2011 and all other statistical analyses were conducted using SAS 9.3. Due to the low number of participants in these categories, age 56–66 and over 66 years old were grouped as 56 years old and over, and “grew up in an agriculture environment” and “professional involvement” were grouped as “involved in agriculture”.

The percentage of participants that correctly answered none, one, two or three questions of the knowledge quiz on the use of antibiotics was calculated. “I do not know” answers were summed with the incorrect answers. The Kruskal–Wallis test was used to evaluate the effect of age, level of education and involvement in agriculture on the knowledge on the use of antibiotics. The percentage of participants that chose each option from the list of most problematic issues in pig production (Q2) was also calculated.

Multinomial logistic regression allows one to predict the odds ratio (ODDS) of the different possible outcomes of a categorically distributed dependent variable, according to a set of independent variables [29]. The multinomial logistic regression model was used to analyze the association between participants’ opinions regarding the most appropriate solution for unhealthy pigs on the farm (Q1), the type of recruitment, socio-demographics and the number of correct answers in the knowledge quiz. Univariate models were built to separately assess the influence of each predictor variable (the type of recruitment, the socio-demographic data and the number of correct answers in the knowledge quiz) on the dependent variables. Predictor variables with *p* < 0.20 were used to build multivariate models. Backward selection was used to eliminate predictor variables until only those with *p* < 0.10 remained in the final model. A similar methodology (models) was applied to evaluate the association between participants’ opinions regarding the consumption of animals housed in “hospital pens” (Q3), regarding the most appropriate solution for unhealthy pigs with different probabilities of recovery after treatment (Q4) and the type of recruitment and the socio-demographic data. Results are presented as ODDS and 95% confidence intervals (95% CI). Statistical associations were reported when *p* < 0.05 and tendency when 0.05 < *p* < 0.1.

## 3. Results

Demographic data are shown in Table 1. The aspects that most influenced the choice of participants when buying meat were price (42%) and food safety (38%), followed by animal welfare (8%), other aspects (8%) and sustainability (4%). Within “other” aspects, participants mentioned organoleptic characteristics (35%), quality of the product and/or the brand (13%), product origin, including country and brand (12%), and nutritional value of animal protein (7%).

### 3.1. Knowledge on Use of Antibiotics

**Figure 1:**
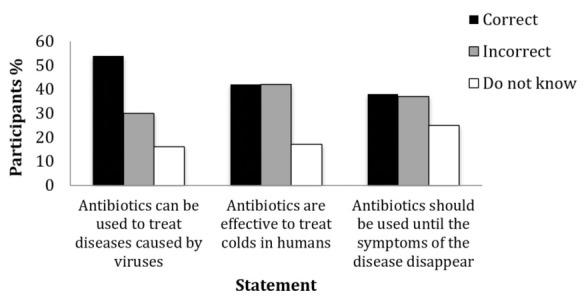
Percentage of correct, incorrect and “I do not know” answers of participants (*n* = 1780) to the three questions of the knowledge quiz on the use of antibiotics.

The percentage of correct, incorrect and “I do not know” answers of participants to the three questions of the knowledge quiz on the use of antibiotics are presented in Figure 1. The percentage of participants that answered one, two or three questions correctly were 17%, 17% and 12%, respectively, and 12% did not answer any question correctly. Age, level of education and involvement in agriculture did not affect the knowledge of participants on the use of antibiotics.

### 3.2. Most Appropriate Solution for Unhealthy Pigs on Farm (Q1)

Just over half of the participants (52%) considered “Moving sick pigs to the “hospital pen” and providing conditions that allow them to improve, including giving antibiotics” as the most appropriate solution for unhealthy pigs on the farm; 36% considered it to be “Euthanizing pigs on the farm at the time of diarrhea or respiratory infection diagnosis to avoid spreading the disease to healthy pigs”, and 13% considered it to be “Moving sick pigs to the “hospital pen”, but not giving antibiotics”.

Participants who were 36–45 and 46–55 years old and those that answered the three questions correctly had lower odds of choosing “Moving sick pigs to the “hospital pen”, but not giving antibiotics” (*p* < 0.05 for both; Table 2). Male participants, those recruited online, and those with higher knowledge on the use of antibiotics also had lower odds of choosing “Moving sick pigs to the “hospital pen” and providing conditions that allow them to improve, including giving antibiotics” (*p* < 0.05; Table 2). In contrast, participants with undergraduate education (completed or on-going) had higher odds of choosing this option than those with up to high school education (*p* < 0.05; Table 2).

### 3.3. Most Problematic Aspect in Pig Production (Q2)

Participants were divided as to the most problematic issue in pig production: “Deficiency in waste management and difficulty in discarding euthanized pigs” (34%), “Inappropriate or excessive use of antibiotics to raise pigs” (32%), and “Suffering of pigs with injuries or disease, which should be immediately euthanized” (31%). Only 3.4% of participants answered “other”.

### 3.4. Approval of Consumption of Animals Housed in “Hospital Pens” (Q3)

Most participants (69%) rejected the consumption of animals housed in “hospital pens”, 17% approved it and 14% were indifferent. Participants that were recruited online, men, those involved in agriculture, those from younger categories, and those that considered meat consumption as important had higher odds of approving the consumption of pigs that were housed in “hospital pens” (*p* < 0.05; Table 3). Participants that were recruited online, older participants and those that had less knowledge on use of antibiotics had lower odds of being indifferent (*p* < 0.05; Table 3). Male participants and those that considered meat consumption as important or indifferent had higher odds of being indifferent (*p* < 0.05; Table 3).

### 3.5. Most Appropriate Solution for Unhealthy Pigs according to Probability of Recovering after Treatment with Antibiotics (Q4)

Regardless of the probability of pigs recovering after treatment with antibiotics, the majority of participants (53%) chose the option “Euthanize this pig on the farm after the disease is diagnosed”, while 41% chose “Applying antibiotics in an effort to recover this animal that will be destined for food production”, and 6% chose “other”. Of the participants that answered “other”, 79% suggested that the most appropriate solution for unhealthy pigs would be to make all efforts to recover the animal, including applying antibiotics, but not using them for food production.

**Figure 2:**
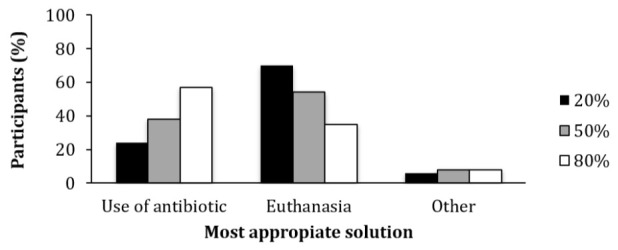
Percentage of participants that chose each of the given options towards the most appropriate solution for unhealthy pigs with different probabilities of recovery (20%, 50% or 80%) after treatment.

Among participants that received the question involving a pig that had a 20% chance of recovering if it is treated with antibiotics, only 24% chose the option “To apply antibiotics in an effort to recover this animal that will be destined for food production”. In contrast, when the pig had an 80% chance of recovering if treated with antibiotics, 57% of participants chose this option (Figure 2).

Table 4 shows that increasing the probability of the pigs recovering after treatment and being involved in agriculture increased the odds of participants choosing the option “Applying antibiotics in an effort to recover this animal that will be destined for food production” (*p* < 0.05). In contrast, participants with higher levels of knowledge on the use of antibiotics (i.e., that answered one or more questions correctly) had lower odds of choosing the option “Applying antibiotics in an effort to recover this animal that will be destined for food production”, compared to those that did not answer any question correctly (*p* < 0.05). Participants that were recruited online and those that were indifferent or considered the consumption of meat as important had, respectively, higher and lower odds of choosing the “other” option (*p* < 0.05).

## 4. Discussion

After reading the information about unhealthy pigs provided in the survey, 52% of participants considered “Moving sick pigs to the “hospital pen” and providing conditions that allow them to improve, including giving antibiotics” as the most appropriate solution for these animals on the farm. This finding was not expected, as citizens have shown concerns about the use of antibiotics in food production [13,16,30] and perceive this practice as a major health risk [31]. Consumers expect that treatments with antibiotics in livestock farming should be reduced and applied very carefully in order to minimize the negative impacts on human health [13]. Increasing the probability of the pigs recovering after treatment was associated with higher odds of participants supporting the use of antibiotics. In general, citizens believe that antibiotics should be used in livestock as a last resort in disease treatment [13,30,32]. In this case, increasing the animals’ chances of recovery was considered an acceptable use of antibiotics. Furthermore, we identified low levels of knowledge on the use of antibiotics in our study population, which is in accordance with previous studies showing that public knowledge about the use and mechanisms of action of antibiotics is very low [13,33,34,35]. This low knowledge could justify the participants’ choice of treating the unhealthy pigs with antibiotics, which contrasts with previous studies reporting consumers’ concerns about the use of antibiotics in food products. This is supported by the fact that participants with higher knowledge on the use of antibiotics did not agree that the use of antibiotics was the most appropriate solution for unhealthy pigs on the farm.

The results from our study seem to highlight the conflict between public concerns towards the use of antibiotics in food production animals and animal welfare aspects related to sick animals, such as health, pain and suffer. Consumers do not seem to be aware that banning or reducing the use of antibiotics in livestock production might have negative repercussions for animal welfare [20], production costs, and an increase in the price of meat [36]. In part, it might be related to the fact that some consumers do not see the value of the use of antibiotics in pain reduction in sick animals [13].

Regardless of the pig’s probability of recovering after treatment with antibiotics, participants involved in agriculture had higher odds of choosing the option that included the use of antibiotics compared to participants not involved in agriculture. This finding could be associated with these participants’ knowledge on the difficulty of ensuring timely euthanasia [37] and that euthanasia of an animal on the farm implies an economic loss for the production system. Caretakers are aware of the welfare aspects (mainly related to pain) of euthanizing sick animals; those working in swine systems have expressed a wish never to have to carry out euthanasia again [38], probably because they feel guilty about performing euthanasia on the farm [27]. Furthermore, the use of antibiotics plays an important role in improving the welfare of livestock animals if used to treat severely sick animals, especially those suffering pain or distress, and pig producers perceived their use as a valuable cost-effective tool to maintain animal health [39]. Unfortunately, farmers appear to have low awareness of the risks of the excessive use of antibiotics in pig husbandry [40,41].

The majority of participants rejected the consumption of meat from animals housed in “hospital pens”, which seems to be associated with concerns for food safety. This has indeed become a major issue of public concern [31,42] and was the second most important factor that influenced our participants when buying meat, after price. Lay citizens are usually unaware of the legislation requiring withdrawal periods for antibiotics in animal production, which contrasts with the knowledge of those involved in agriculture in relation to this issue. In fact, participants involved in agriculture were more likely to support the consumption of pigs that were housed in “hospital pens”, which is within our expectation, as risk perception differs between consumer-citizens and those involved in livestock production [43].

The fact that the majority of participants supported the use of antibiotics and rejected the consumption of meat from animals housed in “hospital pens” is in line with those describing that the most appropriate solution for unhealthy pigs would be to make all efforts to recover the animal, including giving antibiotics, but not using them for food production. This seems to indicate consumers’ concerns regarding animal welfare, which is in accordance with previous studies showing that the general public has become increasingly interested in farm animal welfare and aspects of food animal production, both in developed [11,44] and developing nations [5,8,45,46]. Lay citizens believe that animals are sentient beings with capacity to suffer and have positive emotional states [47], and that to impose pain on animals is unacceptable [48]. Instead, they desire to eat food produced in a way where the animal has not experienced pain [49,50]. Additionally, although the use of antibiotics was not mentioned in the question about participants’ approval of consuming an animal housed in “hospital pens”, rejection of consuming the meat of these animals could suggest that they perceived the risk and “wished” not to eat meat from animals treated with antibiotics [20]. As consumers have little information regarding farm animal practices, most are not aware of the withdrawal periods for antibiotics, and therefore may assume that all meat from treated animals will have residues. Most consumers eat food products from animals treated with antibiotics, but they may not be aware of this [20]. However, their confidence in the food production sector has been decreasing [51] and consumers have increasingly expressed concerns about residues in meat [52], especially hormones and veterinary drug residues [43,46]. Previous studies have already reported that consumers are willing to pay more for antibiotic-free animal products [20,36]. Further studies should address this issue, as it is highly relevant for informing the livestock sector, as it allows adaption for future demands of a population with lower links to rural areas and food production systems.

## 5. Conclusions

In general, for the majority of participants, the most appropriate solution for unhealthy pigs was the use of antibiotics, which was associated with participants’ low knowledge about antibiotics. Moreover, increasing the probability of the pigs recovering after treatment was associated with greater support for the use of antibiotics. However, the majority of participants were not willing to consume animals housed in “hospital pens”. The reason for this was concern with food safety, which was the second most important factor that influenced the participants’ choice when buying meat, after price. Support for the use of antibiotics to deal with unhealthy pigs in “hospital pens”, as well for consumption of the meat from these animals, was higher among participants involved in agriculture. This discrepancy may also be due to the last group having information about regulations mandating withdrawal periods for drugs including antibiotics. The fact that lay participants thought that all efforts should be made to recover the unhealthy pigs, including giving antibiotics, but not using them for food production, indicates a disconnection between consumer expectations and industry practices. It also shows that consumers do not seem to be aware of the potential negative repercussions for animal welfare associated with banning or reducing the use of antibiotics in livestock production, which is an important concern for the industry.

## Figures and Tables

**Table 1:** Demographics of survey participants (*n* = 1780).

Variables	Participants (%)
Type of recruitment	
Face-to-face	28
Online	72
Treatment—probability of animal to recover	
20%	33
50%	35
80%	32
Sex	
Female	56
Male	44
Age	
18 to 25 years old	50
26 to 35 years old	17
36 to 45 years old	10
46 to 55 years old	11
56 years old and over	12
Education	
Up to high school	35
Undergraduate (complete or on going)	65
Involvement in agriculture	
No involvement	85
Involvement	15
For you, consuming meat is …	
Not important	15
Indifferent	37
Important	48
Number of correct answers in the knowledge quiz	
0 answer	12
1 answer	12
2 answers	17
3 answers	17
Do not know (in at least one answer)	42

**Table 2:** Odds ratio (ODDS) and 95% confidence interval (95% CI) for multinomial logistic regression models of opinion towards the most appropriate solution for unhealthy pigs on the farm (Q1). Reference category was: “At the time of diarrhea or respiratory infection diagnosis, euthanize pigs on the farm to avoid spreading the disease to healthy pigs.”

Variables	No Antibiotics ^1^			Antibiotics ^2^		
	ODDS	95% CI		ODDS	95% CI	
Type of recruitment						
						
Online	0.759	0.513	1.122	0.708 *	0.546	0.919
Sex						
Female						
Male	0.943	0.694	1.281	0.714 *	0.580	0.879
Age						
18 to 25 years old						
26 to 35 years old	0.685	0.424	1.106	1.171	0.865	1.585
36 to 45 years old	0.526 *	0.296	0.936	0.853	0.601	1.210
46 to 55 years old	0.439 *	0.243	0.793	0.753	0.528	1.076
56 years old and over	0.864	0.527	1.416	0.844	0.593	1.202
Education						
Up to high school						
Undergraduate	1.045	0.752	1.452	1.353 *	1.078	1.697
Number of correct answers in the knowledge quiz						
0						
1	0.754	0.499	1.140	0.657 *	0.494	0.874
2	0.794	0.525	1.201	0.611 *	0.458	0.814
3	0.487 *	0.293	0.809	0.511 *	0.371	0.703

^1^ “Move sick pigs to the “hospital pen”, but do not give antibiotics”; ^2^ “Move sick pigs to the “hospital pen” and provide conditions that allow them to improve, including giving antibiotics”; * significantly different from the reference category, *p* < 0.05.

**Table 3:** Odds ratio (ODDS) and 95% confidence interval (95% CI) for multinomial logistic regression models of opinion towards approval of humane consumption of animals housed in “hospital pens” (Q3). Reference category was “Reject”.

Variables	Approved			Indifferent		
	ODDS	95% CI		ODDS	95% CI	
Type of recruitment						
Personal						
Online	1.711 *	1.189	2.462	0.657 *	0.467	0.924
Sex						
Female						
Male	1.902 *	1.462	2.474	1.480 *	1.115	1.964
Age						
18 to 25 years old						
26 to 35 years old	0.925	0.646	1.324	0.689	0.454	1.044
36 to 45 years old	0.359 *	0.210	0.613	0.276 *	0.151	0.504
46 to 55 years old	0.489 *	0.293	0.815	0.458 *	0.271	0.774
56 years old and over	0.423 *	0.255	0.700	0.546 *	0.336	0.887
Involvement in agriculture						
No involvement						
Involvement	2.052 *	1.476	2.854	1.073	0.710	1.622
For you, consuming meat is…						
Not important						
Indifferent	2.690 *	1.756	4.121	3.874 *	2.129	7.049
Important	1.405	0.888	2.222	3.314*	1.809	6.073
Number of correct answers in the knowledge quiz						
0						
1	1.052	0.726	1.524	0.796	0.556	1.140
2	1.284	0.892	1.848	0.568 *	0.379	0.850
3	1.272	0.842	1.921	0.838	0.546	1.285

* Significantly different from the reference category, *p* < 0.05.

**Table 4:** Odds ratio (ODDS) and 95% confidence interval (95% CI) for multinomial logistic regression models of opinion towards the most appropriate solution for unhealthy pigs with different probabilities of recovery after treatment (Q4). The reference category was “To euthanize this pig on the farm after the disease is diagnosed”.

Variables	Antibiotics ^1^			Other		
	ODDS	95% CI		ODDS	95% CI	
Type of recruitment						
**Personal**						
**Online**	0.970	0.772	1.219	2.086 *	1.185	3.670
Treatment—probability of animal to recover						
20%						
50%	2.140 *	1.663	2.752	1.619	0.957	2.737
80%	4.563 *	3.521	5.913	2.988 *	1.768	5.049
Involvement in agriculture						
No involvement						
Involvement	1.454 *	1.100	1.922	0.556	0.261	1.184
For you, consuming meat is …						
Not important						
Indifferent	1.298	0.950	1.773	0.385 *	0.230	0.645
Important	1.055	0.762	1.459	0.378 *	0.221	0.648
Number of correct answers in the knowledge quiz						
0						
1	0.661 *	0.502	0.870	0.736	0.418	1.295
2	0.717 *	0.544	0.946	0.677	0.379	1.209
3	0.567 *	0.412	0.781	0.815	0.437	1.519

^1^ “To give antibiotics in an effort to recover this animal that will be destined for food production”; * significantly different from the reference category, *p* < 0.05.
